# Items

**Published:** 1901-05

**Authors:** 


### ITEMS.

Messrs. Wm. R. Warner & Co., pharmaceutical chemists, of Philadelphia, announce that their business will be continued substantially as it was conducted during the life-time of Mr. Warner, senior, and without interruption. The firm name, William R. Warner & Co., will remain unchanged. The standard of excellence of their products will be scrupulously maintained and all who have had transactions with them can rely upon their statement, that the past will foreshadow the future in every detail.

---

Physicians in active practice will be interested in reading the advertisement, on page xxv., relating to Duffy's Malt Whiskey. When alcoholics are needed in the treatment of disease it is of the first importance that they shall be pure. This condition is fully met in the Duffy Malt Whiskey.

---

Wyeth's prepared food, the maker's claim, is not a mere mechanical mixture, the bulk of which is composed of sugar; in fact, it contains no cane sugar, glucose, molasses or sweetener of any kind, its pleasant taste rendering it so acceptable to invalids, infants and others, being derived entirely from the dessicated milk, with its natural sugar of milk, combined with the agreeable odor of the prepared cereals and the sweetness of the extract of malt. They also state that in infant's food it is positively ideal, as more nutritious and at the same time simpler materials for such a food could not, in their judgment, be brought together; and by mixing it with fresh, pure milk, and in some cases a little cream, a most nourishing and powerfully restorative food can be obtained for older children or adults.

